# Opposite Effect of Thyroid Hormones on Oxidative Stress and on Mitochondrial Respiration in COVID-19 Patients

**DOI:** 10.3390/antiox11101998

**Published:** 2022-10-08

**Authors:** Claudia De Vitis, Carlo Capalbo, Alessandra Torsello, Christian Napoli, Valentina Salvati, Chiara Loffredo, Giovanni Blandino, Giulia Piaggio, Francesca Romana Auciello, Flaminia Pelliccia, Gerardo Salerno, Maurizio Simmaco, Laura Di Magno, Gianluca Canettieri, Flaminia Coluzzi, Rita Mancini, Monica Rocco, Salvatore Sciacchitano

**Affiliations:** 1 Department of Clinical and Molecular Medicine, Sapienza University of Rome, 00189 Rome, Italy; 2 Department of Medical Oncology, Sant’Andrea University Hospital, 00189 Rome, Italy; 3 Department of Molecular Medicine, Sapienza University of Rome, 00189 Rome, Italy; 4 Department of Surgical and Medical Science and Translational Medicine, Sapienza University of Rome, 00181 Rome, Italy; 5 Scientific Direction, IRCCS Regina Elena National Cancer Institute, 00144 Rome, Italy; 6 Unit of Anesthesia, Intensive Care and Pain Medicine, Sant’Andrea University Hospital, 00189 Rome, Italy; 7 Translational Oncology Research Unit, IRCCS Regina Elena National Cancer Institute, 00144 Rome, Italy; 8 UOSD SAFU, Department of Research, Diagnosis and Innovative Technologies, IRCCS Regina Elena National Cancer Institute, 00144 Roma, Italy; 9 Department of Neuroscience, Mental Health, and Sensory Organs (NESMOS), Sapienza University of Rome, 00189 Rome, Italy; 10 Pasteur Institute, Cenci-Bolognetti Foundation, 00161 Rome, Italy; 11 Department Medical and Surgical Sciences and Biotechnologies, Sapienza University of Rome, Polo Pontino, 04100 Latina, Italy

**Keywords:** thyroid hormones (THs), nonthyroidal illness syndrome (NTIS), COVID-19, bioelectrical impedance analysis (BIA), reactive oxygen species (ROS), biological antioxidant potential (BAP), oxidative stress, extracellular flux analyzer, mitochondrial respiration

## Abstract

Background: Thyroid hormones (TH)s are master regulators of mitochondrial activity and biogenesis. Nonthyroidal illness syndrome (NTIS) is generally considered an adaptative response to reduced energy that is secondary to critical illness, including COVID-19. COVID-19 has been associated with profound changes in the cell energy metabolism, especially in the cells of the immune system, with a central role played by the mitochondria, considered the power units of every cell. Infection by severe acute respiratory syndrome coronavirus 2 (SARS-CoV-2) affects and alters mitochondrial functions, both to influence its intracellular survival and to evade host immunity. Aim of the study: This study was undertaken to analyze the oxidative balance and mitochondrial respiration in COVID-19 patients with and without NTIS to elucidate the role that thyroid hormones (TH)s play in this context. Methods: In our cohort of 54 COVID-19 patients, admitted to our University Hospital during the COVID-19 pandemic, we evaluated the generation of reactive oxygen species (ROS) by measuring the serum levels of derivatives of reactive oxygen metabolites (dROMs), and we analyzed the antioxidant capacity by measuring the serum biological antioxidant potential (BAP). We then analyzed the mitochondrial respiration in peripheral blood mononuclear cells (PBMC)s of 28 of our COVID-19 patients, using the seahorse instrument (Agilent). Results were correlated with the serum levels of THs and, in particular, of FT3. In addition, the role of T3 on bioelectrical impedance analysis (BIA) and mitochondrial respiration parameters was directly evaluated in two COVID-19 patients with NTIS, in which treatment with synthetic liothyronine (LT3) was given both in vivo and in vitro. Results: In our COVID-19 patients with NTIS, the dROMs values were significantly lower and the BAP values were significantly higher. Consequently, the oxidative stress index (OSi), measured as BAP/dROMs ratio was reduced compared to that observed in COVID-19 patients without NTIS, indicating a protective role exerted by NTIS on oxidative stress. In our COVID-19 patients, the mitochondrial respiration, measured in PBMCs, was reduced compared to healthy controls. Those with NTIS showed a reduced maximal respiratory capacity and a reduced proton leak, compared to those with normal FT3 serum values. Such lowered mitochondrial respiratory capacity makes the cells more vulnerable to bioenergetic exhaustion. In a pilot study involving two COVID-19 patients with NTIS, we could reinforce our previous observation regarding the role of T3 in the maintenance of adequate peripheral hydroelectrolytic balance. In addition, in these two patients, we demonstrated that by treating their PBMCs with LT3, both in vitro and in vivo, all mitochondrial respiration parameters significantly increased. Conclusions: Our results regarding the reduction in the serum levels of the reactive oxygen species (ROS) of COVID-19 patients with NTIS support the hypothesis that NTIS could represent an adaptative response to severe COVID-19. However, beside this beneficial effect, we demonstrate that, in the presence of an acute reduction of FT3 serum levels, the mitochondrial respiration is greatly impaired, with a consequent establishment of a hypoenergetic state of the immune cells that may hamper their capacity to react to massive viral infection.

## 1. Introduction

Nonthyroidal illness syndrome (NTIS) is generally considered part of the adaptive host neuroendocrine and metabolic response to survive critical illness [1]. It can be diagnosed in up to 70% of critically ill patients [2,3,4,5] of all ages [6]. In the International Classification of Diseases, 11th revision, NTIS has a diagnostic code (5A06), under the old name of sick-euthyroid syndrome [7], and is classified among the disorders of the thyroid gland or of the thyroid hormones (TH)s systems. NTIS is observed in intensive care units (ICU) in association with many different conditions and diseases and has a negative prognostic impact on the course of the disease, with a relevant increased risk of death [8,9,10,11]. However, even if the prognostic relevance of reduced FT3 serum levels is clearly demonstrated, the treatment of NTIS with either synthetic liothyronine (LT3) or levothyroxine (LT4) is still debated [12]. Many of the randomized clinical trials performed so far failed to clearly demonstrate any beneficial effect of such treatments. Recently, we postulated that the lack of convincing evidence reported by many published interventional randomized clinical trials might rely on the choice of inadequate primary outcomes [13]. In the absence of any clear evidence regarding the beneficial effect of the treatment on THs in such patients, the current opinion is that such treatment should not be given unless patients show clear clinical signs of hypothyroidism. Even the assessment of thyroid function in seriously ill patients should not be performed except when there is a strong suspicion of thyroid dysfunction [14]. This is also the opinion of the experts in the thyroid field, as stated in the guidelines published by the American Thyroid Association [15]. 

NTIS has been detected in COVID-19 patients too and, as for other critical conditions, its occurrence in such patients is associated with a higher risk of a more severe disease [16] as well as of death [17,18]. We have recently demonstrated that in COVID-19 patients, the acute deficiency of T3, typically observed in NTIS, is responsible for the occurrence of hydroelectrolytic disequilibrium at the peripheral level, with the induction of an anasarcatic condition, similar to that observed in myxedema, due to long standing, untreated, overt hypothyroidism, which can be easily measured by bioelectrical impedance analysis (BIA) [17]. In addition, by means of the nanostring analysis, we demonstrated that reduced levels of FT3 were associated with the dysregulation of many genes coding for protein located in the mitochondria or involved in mitochondrial function [16].

Mitochondria play pivotal roles in cellular energy metabolism. Indeed, they supply most of the intracellular adenosine triphosphate (ATP), the organic compound that provides energy to drive many processes in every single living cell. For this reason, the analysis of mitochondrial dysfunctions performed in PBMCs has gained much attention. In fact, this cell population mirrors systemic changes within the body and, for this reason, provides a source of sensitive peripheral biomarkers in several diseases [19], including COVID-19. Mitochondria are thought to play a central role in the immune response to viruses and it has been suggested that the maintenance of mitochondrial integrity is essential, especially for an adequate innate immune system response to SARS-CoV-2 virus infection [20]. The inflammatory state induced by the SARS-CoV-2 virus infection, and known as “cytokine storm”, is associated with the elevated production of reactive oxygen species (ROS), responsible for the induction of oxidative stress. Both alterations point toward an altered mitochondrial activity in patients affected by COVID-19 [21]. Moreover, the debilitating condition consisting of the occurrence of redox imbalance, energy metabolic deficits, and a hypometabolic state, observed in the post-acute sequelae of severe acute respiratory syndrome coronavirus 2 infection (PASC), has been linked to mitochondrial damage [22,23] and to mitochondrial dysfunction of any cause [24]. Mitochondria represent one of the major ROS sources and the occurrence of altered mitochondrial dynamics is crucial in the development of the disease. For this reason, the analysis of these cellular organelles and of the substances that are directly involved in the regulation of oxidative stress is essential. 

Thyroid hormones (TH)s are master regulators of cell metabolism [25]. They regulate a variety of pathways involved in metabolism and energy expenditure (i.e., burned calories), both in the resting metabolic rate and during physical activity [26]. Their role in stimulating cell energy has been known for long time [27]. It consists of a relative acceleration of the basal metabolism that includes an increase in the rate of both catabolic and anabolic reactions. As a consequence of such a stimulatory effect on cell metabolism, they produce an increase in ROS generation. Many of their effects are exerted on metabolically active organs, including the liver, brown adipose tissue (BAT), white adipose tissue (WAT), skeletal muscle, and the heart. In brown adipocytes, they enhance basal, maximal, ATP-linked, and proton-leak oxygen consumption rates (OCR)s. In these cells, they dissipate the generated energy via heat production by proton currents and by means of the uncoupling protein 1 (UCP-1) [28]. However, their action has also been studied in innate immune cells, and remarkable and crucial effects on neutrophils, macrophages, and dendritic cells have been reported [29]. It has been demonstrated that THs participate to the mechanism of inflammation and to oxidative stress [30]. Their role in the maintenance of an adequate level of energy in immune cells as well as the effects of acute deficiency of FT3 on mitochondrial respiration and on oxidative stress at the periphery in COVID-19 patients has not yet been examined. The aim of this study was to investigate the effects of acute deficiency of FT3 on the oxidative balance by measuring the derivatives of reactive oxygen metabolites (dROMs) and the biological antioxidant potential (BAP) in the serum and by analyzing the mitochondrial respiration in the PBMCs obtained from a cohort of COVID-19 patients with and without NTIS. 

## 2. Methods

### 2.1. Study Group

During the last COVID-19 pandemic waves, we collected data and blood samples from a total of 54 patients admitted to the ICU of our University Hospital. In 44 of them, the FT3 serum levels were low (≤1.7 pg/mL), with TSH serum levels reduced or inappropriately normal, in the context of a nonthyroidal illness syndrome (NTIS). In the remaining 10 patients, the serum levels of FT3 were normal (>1.8 pg/mL). Patients with low FT3 serum values were further subdivided into two groups, 9 with very low FT3 (≤1.0 pg/mL) and 35 with moderately low FT3 serum values (>1.0 pg/mL and ≤1.7 pg/mL). The epidemiological and clinical data of the patients included in the study are reported in Table 1. In our COVID-19 patients, we observed a much higher occurrence of comorbidities among those that presented low or very low FT3 serum levels. In particular, we observed a higher frequency of cardiovascular disease and hypertension.

Patients were examined at admission. Serums taken from all patients were used to measure TH function and to assess oxidative stress. At the same time, in 28 of them (20 with low and 8 with normal FT3 serum values), we collected PBMCs to perform in vitro analysis of mitochondrial respiration.

### 2.2. Thyroid Hormone Function Tests

Thyroid hormone measurements were performed using a Chemiluminescent Microparticle Immunoassay (CMIA), an immunoassay analyzer (ARCHITECT i1000SR, Abbott Lab., Abbott Park, IL, USA), and specific, dedicated diagnostic kits (ARCHITECT Free T3, FT4 and TSH assay, Abbott Lab., Abbott Park, IL, USA), as previously described [16]. The conventional reference intervals for FT3, FT4, and TSH were 1.71–3.71 pg/mL, 0.7–1.48 ng/dL and 0.35–4.0 µIU/mL, respectively.

### 2.3. Lethality

We registered and reported lethality of COVID-19 patients, hospitalized in the ICU during the study period, and we correlated it with the FT3 serum levels measured at admission.

### 2.4. Measurement of the Reactive Oxygen Species (ROS) and of Biological Antioxidant Potential (BAP) in Serum of COVID-19 Patients with NTIS

Blood samples were collected from COVID-19 patients and immediately centrifuged at 3000 rpm for 10 min to separate the serum. The separated serum samples were stored in a freezer at −20 °C until assay. The measurement of the dROMs was performed on the blood of our COVID-19 patients using the colorimetric dROMs test (Diacron srl, Grosseto, Italy), as previously described [31]. The results were expressed in Carratelli Units (U CARR). A single U CARR is equivalent to the H_2_O_2_ of 0.08 mg/dL. Reference values were the following: 300–320 U CARR border line values; 321–340 U CARR mild oxidative stress; 341–400 U CARR moderate oxidative stress; 401–500 U CARR high oxidative stress; >500 U CARR severe oxidative stress.

Measurements of BAP were performed on the blood of our COVID-19 patients using the colorimetric BAP test (Diacron srl, Grosseto, Italy), as previously described [31]. The results were expressed in μmol/L. Reference values were the following: >2200 μmol/L optimal level; 2000–2200 μmol/L borderline values; 1800–2000 μmol/L mild deficiency; 1600–1800 μmol/L deficiency; <1600 μmol/L severe deficiency. Both tests were measured using the same serum sample and the same testing equipment (Libra S12 luminometer, Biochrom, Cambridge, UK). The oxidative stress for each sample was calculated by the ratio of the dROMs test result, assumed as a measure of the oxidation degree, to the BAP test result, indicative of the antioxidant capacity of the tested serum and expressed as oxidative stress index (OSi).

### 2.5. Isolation of PBMC from COVID-19 Patients with NTIS

We isolated the peripheral blood mononuclear cells (PBMC)s from the blood samples of our COVID-19 patients, as previously described [16]. PBMCs were immediately frozen and maintained in the Biobank at the Regina Elena Cancer Institute (BBIRE). To perform the analysis, the PBMCs were retrieved from the Biobank, thawed, and an aliquot was used to perform the analysis of oxidative stress and functional metabolic assessment. As a control, we used the PBMCs of three healthy donors.

### 2.6. Assessment of Mitochondrial Respiration

We analyzed the two major energy pathways of the cell, namely the oxidative phosphorylation and the glycolysis in the PBMCs, of our COVID-19 patients. Measurements were performed using the XFe-96 Extracellular Flux Analyzer (Seahorse Bioscience, Agilent, Santa Clara, CA, USA). Oxidative phosphorylation was measured by OCR and glycolysis, by the extracellular acidification rate (ECAR). PBMCs were seeded in an XF medium in the assay wells of the custom 96-well XF miniplate at a confluency of 50–90%. Cells were incubated in a CO_2_-free incubator to allow equilibration prior to loading into the XF-96 apparatus. Perturbation profiling of the mitochondrial respiration was achieved using the Seahorse XF Cell Mito Stress Test Kit (#103015-100, Agilent). After measuring the initial OCR, 1 μM oligomycin was added to inhibit the ATP synthesis from oxidative phosphorylation. Then, 1 μM carbonyl cyanide-4-(trifluoro-methoxy) phenylhydrazone (FCCP) was added to uncouple the mitochondrial membrane that stimulates respiration. Lastly, 1 μM rotenone and 1 μM antimycin A (R + A) were added to inhibit complex I and III, which terminates mitochondrial oxidative phosphorylation. The basal OCR was calculated as [OCR_initial_ − OCR_R+A_]. The maximum respiration rate was computed as [OCR_FCCP_ − OCR_R+A_].

### 2.7. Assessment of BIA and of Mitochondrial Respiration in Two COVID-19 Patients with NTIS

In two patients affected by COVID-19, both presenting NTIS, we investigated the effect of LT3 treatment on bioelectrical impedance analysis (BIA) parameters and on mitochondrial respiration. The patients’ clinical data are reported in Table 2.

Body composition analysis of our two patients was performed using a single-frequency bioelectrical impedance analysis (SF-BIA), namely the BIA 101 analyzer (Akern Srl, Pontassieve, Firenze, Italy), as previously described [17]. We measured three parameters, namely the resistance (Rz), the reactance (Xc), and the phase angle (PhA). Based on these parameters, we obtained estimates of a number of BIA parameters, including the body cell mass (BCM), the total body water (TBW), the extracellular water (ECW), the intracellular water (ICW), the Na:K exchange rate (Na*_e_*:K*_e_*), the fat-free mass (FFM), and the fat mass (FM). In addition, we calculated the hydration and nutrition state, using the following formulas: hydration = TBW/FFM and nutrition = mg/24 h/htm [The BIA compendium, 3 (http://www.data-input.de, accessed on 20 August 2022)]. BIA analysis was performed on both patients at T0 and T1, using the Bodygram PRO software (vers. 3.0, Jatreia, Pescantina, Verona, Italy).

Mitochondrial respiration analysis was measured in patient 1 on PBMCs isolated at T0 in the absence or in the presence of LT3, added in vitro at a final concentration of 100 nM for 1 h. In patient 2, measurements were performed on PBMCs at T0 and at T1 after in vivo treatment of the patient with LT3, given orally at 63.9 µg/die for one week.

### 2.8. Statistical Analysis

Continuous variables (quantitative) are described by mean and standard deviation (SD), while categorical variables are described by frequency or percentage. The comparison of quantitative variables between groups was performed using the Wilcoxon test, Mann–Whitney test and ANOVA test. Descriptive statistical analysis was performed on raw data where applicable. Results were expressed as means ± SD. A two-tailed *p* value of 0.05 or less was used as a criterion to indicate statistical significance. NS = not significant. Data were statistically analyzed using the GraphPad Prism software (vers. GP9-2273399-RKSP-6761C) (GraphPad Software, San Diego, CA, USA).

### 2.9. Ethics 

The study was approved by the Institutional Ethical Committee of our University La Sapienza of Rome, Italy (RIF. CE 5773_2020, Prot. # 52SA_2020, and its subsequent substantial amendment RIF. CE 5773_2020, Prot. # 171SA_2020), on the basis that it complied with the Declaration of Helsinki, that the protocol followed existing good clinical practice guidelines, and that informed consent was obtained from each individual.

## 3. Results

### 3.1. Lethality

In our COVID-19 patients, lethality was associated with the occurrence of low FT3 serum values. During the study period, 14 patients died during hospitalization, an overall mortality rate of 25.9%. The lethality rate was higher among the group of COVID-19 patients with low FT3 serum values at admission (29.5%), compared to that registered among COVID-19 patients that showed normal FT3 serum values at admission (10.0%) (Supplementary Figure S1). It is interesting to note that, while patients with normal FT3 serum values who died during hospitalization showed no comorbidity, all patients that died in the group of low or very low FT3 serum values presented at least one comorbidity, namely hypertension or cardiovascular disease, suggesting a more severe and complicated disease. However, we were not able to report the exact cause of death for each of them. 

### 3.2. Generation of Reactive Oxygen Species (ROS) in COVID-19 Patients with NTIS 

**Figure 1:**
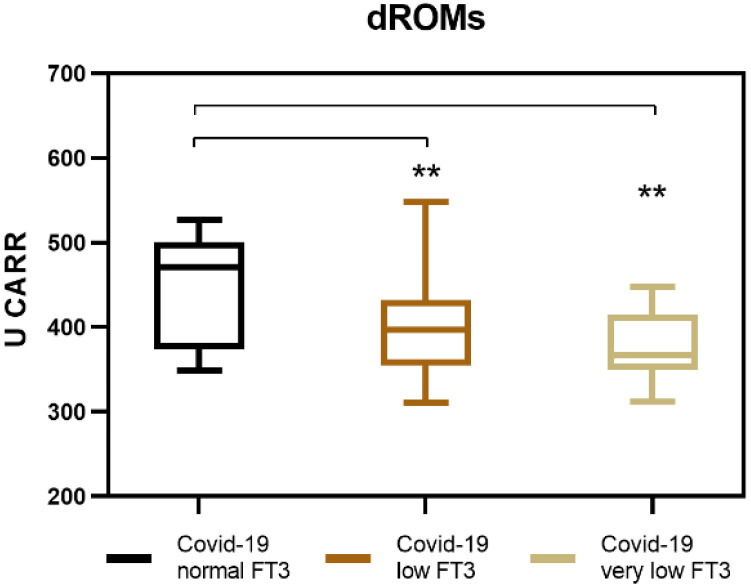
Measurement of reactive oxygen species (ROS) in COVID-19 patients with NTIS. ** *p* < 0.005.

In COVID-19 patients with NTIS, we observed a reduced generation of ROS compared to COVID-19 patients without NTIS (Figure 1). In particular, those with very low levels of serum FT3 (≤1.0 pg/mL) showed significantly lower medium levels of dROMs (376.5 U CARR) compared to those with moderately low FT3 serum levels (>1.0 ≤1.7 pg/mL) that showed higher medium levels of dROMs (395 U CARR). The difference was still remarkable when dROMs values were considered in these two groups of patients combined (all with FT3 serum levels ≤ 1.7 pg/mL) and were compared to those obtained in COVID-19 patients with normal FT3 serum levels (>1.8 pg/mL). These patients showed the highest medium dROMs values observed (449.5 U CARR) (Table 3). These results indicate that the generation of ROS is dependent on the presence of FT3.

### 3.3. Biological Antioxidant Potential (BAP) in COVID-19 Patients with NTIS

**Figure 2:**
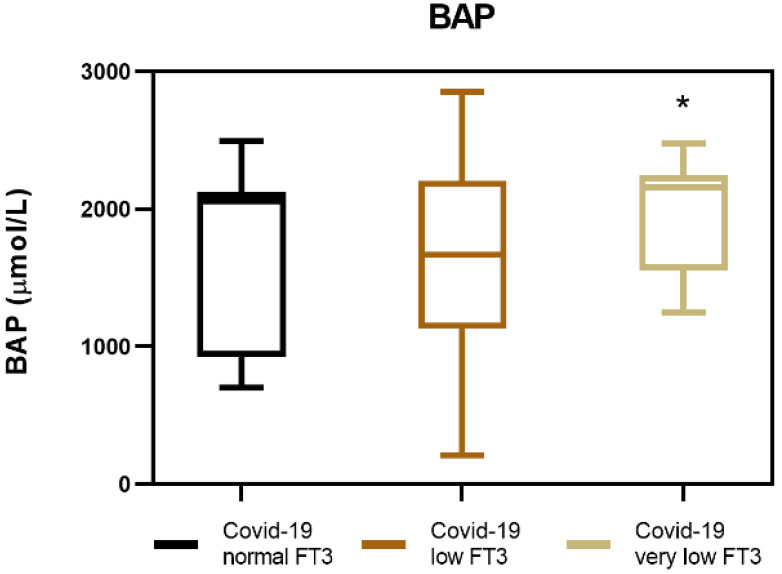
Measurement of biological antioxidant potential (BAP) in COVID-19 patients with NTIS. * *p* < 0.05.

Since THs are known to have effects on both oxidation and antioxidation processes, we tested the serum of our COVID-19 patients for their ability to reduce Fe^3+^ ions to Fe^2+^ ferrous ions in the BAP test. The results of the BAP test indicate that COVID-19 patients with NTIS, and especially those that have very low FT3 serum levels, have a higher medium antioxidant potential (2068.4 µmol/L) when compared to COVID-19 patients that do not have NTIS (1605.4 µmol/L) (Figure 2, Table 3). 

### 3.4. Oxidative Stress Index (OSi) in COVID-19 Patients with NTIS 

The occurrence of oxidative stress is due to the imbalance between the production and accumulation of free radicals and the existing antioxidant capacity of the host serum. Therefore, we measured the balance between the oxidative state and the antioxidant capacity. To this purpose, we calculated the OSi in our patients as an oxidant to antioxidant (dROMs/BAP) ratio. We found that COVID-19 patients, especially those with very low levels of serum FT3, have significantly reduced medium levels of OSi (0.19) compared to those with normal FT3 serum levels (0.33) (Table 3).

Taken together, the results of the dROMs, the BAP tests, and the OSi indicate that the reduction of FT3 serum levels have a protective role on the oxidative stress in COVID-19 patients, confirming the hypothesis that NTIS represents a beneficial adaptive response to the acute viral infection. 

### 3.5. Mitochondrial Respiration in the PBMCs of COVID-19 Patients. Effects of FT3 Serum Levels

Compared to healthy controls, COVID-19 patients showed a marked reduction in the kinetics of mitochondrial respiration (Supplementary Figure S2). 

**Figure 3:**
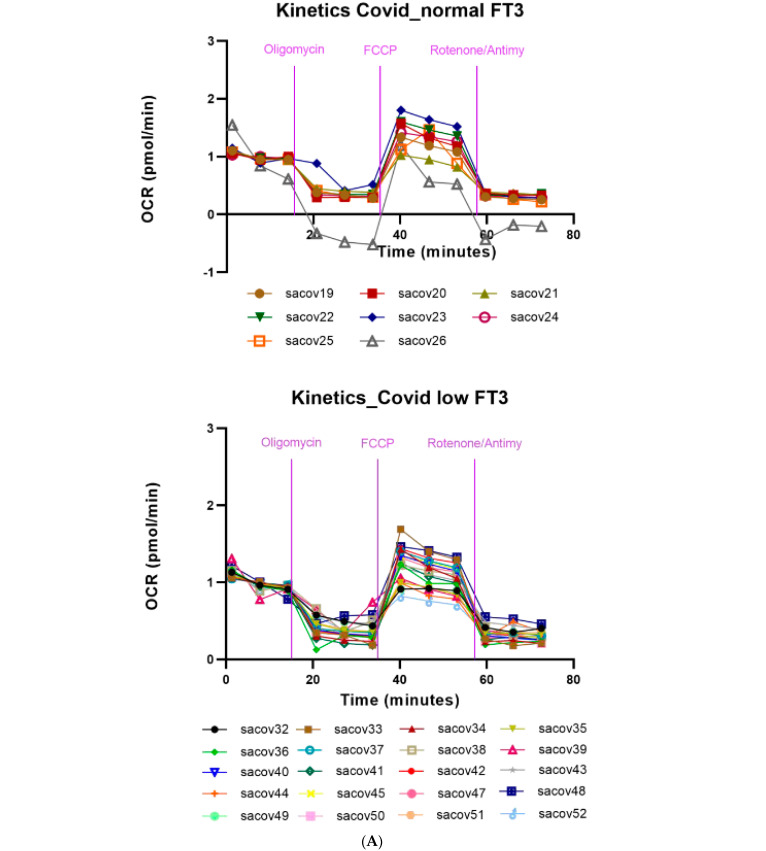

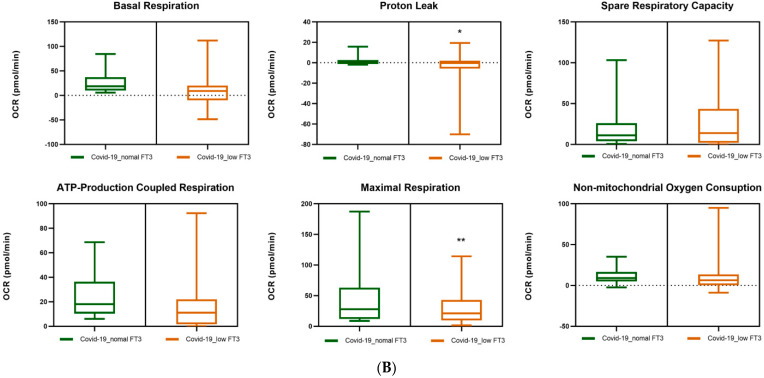
(**A**) Mitochondrial respiration in COVID-19 patients with (upper panel) and w/o (lower panel) NTIS. Traces of OCR of PBMCs from each single COVID-19 patient are reported. (**B**) Mitochondrial respiration in COVID-19 patients with and w/o NTIS. Measurement of basal respiration, proton leak, spare respiratory capacity, ATP-production coupled respiration, maximal respiration, and non-mitochondrial oxygen capacity of PBMCs from COVID-19 patients with and w/o NTIS. Data represent individual values, mean, and standard error of the mean (CTR, *n* = 12; COVID, *n* = 13). The Wilcoxon test was used for statistical analysis. Exact *p* values are reported in the figure. * *p* < 0.05; ** *p* < 0.005.

In particular, the PBMCs obtained from COVID-19 patients showed a reduction in all parameters of mitochondrial respiration, examined by Seahorse, with a remarkable reduction in both basal and maximal respiration capacity. We then evaluated the effects of the reduced levels of serum FT3 in the PBMCs obtained from our cohort of COVID-19 patients, divided between those that presented low FT3 serum levels (≤1.7 pg/mL) and those that showed normal FT3 serum levels (>1.8 pg/mL). No significant differences were observed in the two groups of PBMC samples with regards to basal respiration, ATP-linked respiration, spare capacity, and non-mitochondrial oxygen consumption. PBMCs of COVID-19 patients with low FT3 showed a significant reduction in the medium values of maximal mitochondrial respiration and in the proton leak, compared to those with normal FT3 (Figure 3A,B).

Both these parameters of mitochondrial respiration were reduced in COVID-19 patients with low FT3 serum levels, in the context of NTIS. Low FT3 serum levels, therefore, appear to be associated with a reduced respiration ability to operate at maximum respiratory capacity and to respond to relevant stress conditions. T3-dependent reduced the ability to properly adapt to stress conditions, exposing the cell to an inability to ensure adequate bioenergetic levels to meet the increasing needs for an efficient immune response to the SARS-CoV-2 infection.

### 3.6. Effects of In Vitro and In Vivo LT3 Treatment in Two COVID-19 Patients with NTIS 

In the attempt to confirm our results, obtained in the cohort of COVID-19 patients, and to demonstrate the specific role of LT3 treatment in ameliorating the mitochondrial respiration, we performed a pilot study using the PBMCs obtained from two COVID-19 patients, who showed low FT3 serum levels at admission. In addition, we performed BIA at T0 and T1 in both patients. Mitochondrial respiration analysis was repeated in patient 1 after in vitro treatment of PBMCs with LT3, and in patient 2, after in vivo treatment with LT3. The clinical data of the two patients are reported in Table 2. 

**Figure 4:**
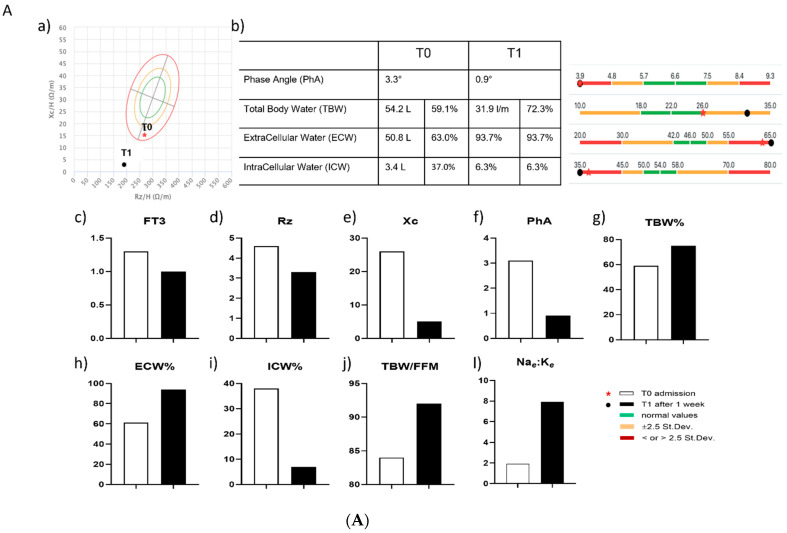

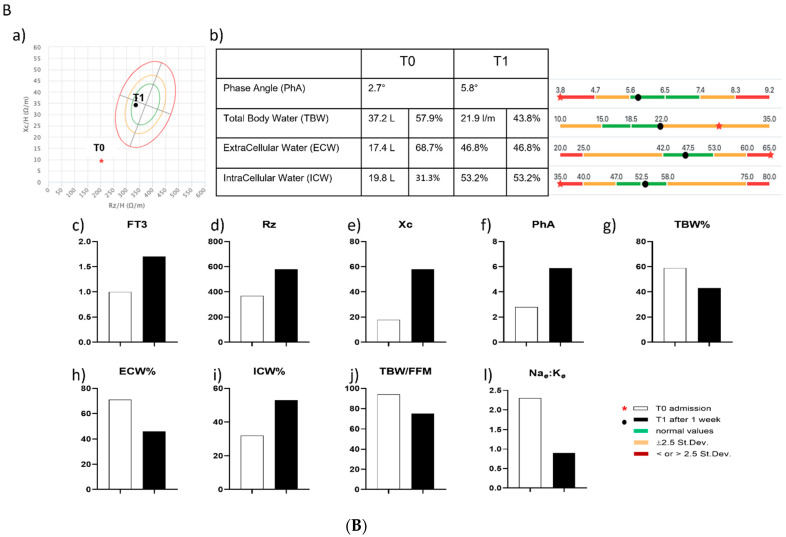
(**A**) BIA measurements in one COVID-19 patient (patient 1) in the pilot study. Measurements performed in a single patient (patient 1), affected by COVID-19, and presenting NTIS at admission, with very low FT3 serum values that were further reduced after one week. (**a**) Rz-Xc (resistance versus reactance) graph, the red star indicates measurement at admission while the black circle indicates measurement after one week; BIA parameters at T0—admission (white bars) and at T1—after one week (black bars); (**b**) graphical representation of the following BIA parameters: PhA, TBW, ECW, and ICW, expressed as % of body weight, measured at T0 and T1; green bars indicate normal reference values, orange bars indicate values included between − and +2.5 standard deviation, red bars indicate values outside– and + 2.5 standard deviation; (**c**) serum FT3 levels in pg/mL, measured at admission (T0) and after one week (T1); changes between T0 and T1 are reported for the following BIA parameters: (**d**) Rz in ohms (Ω); (**e**) Xc in ohms (Ω); (**f**) PhA in degrees (°); (**g**) TBW %, percentage of total body water; (**h**) ECW %, percentage of extracellular water; (**i**) ICW %, percentage of intracellular water; (**j**) TBW/FFM ratio; (**l**) Na*_e_*:K*_e_* ratio, sodium/potassium exchangeable ratio. (**B**) BIA measurements in one COVID-19 patient (patient 2) in the pilot study. Measurements performed in a single patient (patient 2), affected by COVID-19, and presenting NTIS at admission, with very low FT3 serum values that were corrected with LT3 administration at 63.9 µg/die for one week. (**a**) Rz-Xc (resistance versus reactance) graph, the red star indicates measurement at admission while the black circle indicates measurement after one week; BIA parameters at T0—admission (white bars) and at T1—after one week (black bars). (**b**) graphical representation of the following BIA parameters: PhA, TBW, ECW, and ICW, expressed as % of bodyweight, measured at T0 and T1; green bars indicate normal reference values, orange bars indicate values included between − and +2.5 standard deviation, red bars indicate values outside– and +2.5 standard deviation; (**c**) serum FT3 levels in pg/mL, measured at admission (T0) and after one week (T1); changes between T0 and T1 are reported for the following BIA parameters: (**d**) Rz in ohms (Ω); (**e**) Xc in ohms (Ω); (**f**) PhA in degrees (°); (**g**) TBW %, percentage of total body water; (**h**) ECW %, percentage of extracellular water; (**i**) ICW %, percentage of intracellular water; (**j**) TBW/FFM ratio; and (**l**) Na*_e_*:K*_e_* ratio, sodium/potassium exchangeable ratio.

Patient 1, at admission (T0), had a BMI of 26, a SOFA score of 5, and low FT3 serum levels (1.3 pg/mL), associated with low TSH serum levels (0.16 µIU/mL). After one week (T1), FT3 dropped rapidly, and it was measured at a very low level (less than 1.0 pg/mL) (Table 2). All BIA parameters, including Rz, Xc, PhA, TBW%, ECW%, ICW%, TBW/FFM, and the Na*_e_*:K*_e_* ratio dramatically worsened (Figure 4A). In particular, Rz, Xc, and PhA, were significantly reduced, while TBW%, ECW%, and the TBW/FFM ratio were increased. The ICW% dropped dramatically. Finally, the Na*_e_*:K*_e_* ratio showed a seven-fold increase compared to the level registered at time T0 (Figure 4A). The patient died after a few days.

Patient 2, at admission (T0), had a BMI of 29.4, a SOFA score of 2, and a very low FT3 serum level (<1.0 pg/mL), associated with low TSH serum levels (0.39 µIU/mL) (Table 2). This patient received treatment with oral LT3 (Liotir, IBSA, 1 drop = 0.71 µg) for one week at a dosage of 30 drops (equal to 21.3 µg) TID, for a total of 63.9 µg/die. BIA parameters, including Rz, Xc, PhA, TBW%, ECW%, ICW%, TBW/FFM, and the Na*_e_*:K*_e_* ratio dramatically improved and returned to normal values (Figure 4B). In particular, the Rz, Xc, and PhA, were all increased, the TBW%, the ECW%, and the TBW/FFM ratio were reduced, the ICW% showed a near two-fold increase, and the Na*_e_*:K*_e_* ratio showed a three-fold reduction, compared to the level registered at time T0. The patient continued receiving LT3 treatment until she was dismissed by the hospital.

**Figure 5:**
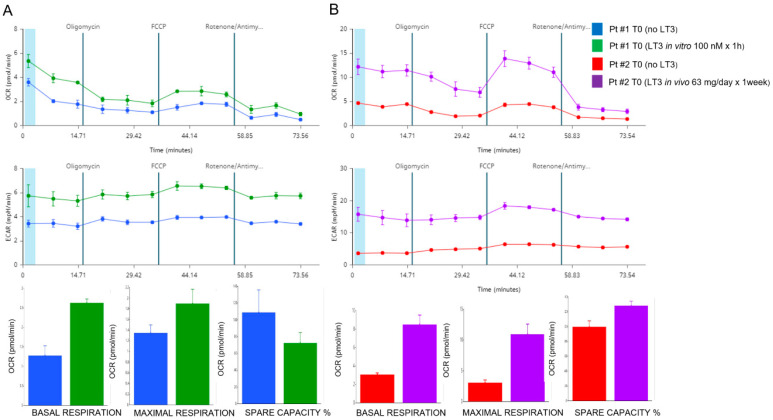
Mitochondrial respiration in two COVID-19 patients in the pilot study. Seahorse analysis of oxygen consumption rate (OCR) and extracellular acidification rate (ECAR) performed on PBMCs obtained from COVID-19 patients with NTIS and treated in vivo and in vitro with LT3. (**A**) The analysis of the PBMCs from patient 1 before (in blue) and after (in green) in vitro treatment with 100 nM of LT3 for 1 h. The profiles of oxygen consumption and extracellular acidification rates are reported in the upper panels; the graphs showing basal and maximal OCR and spare capacity are reported in the lower panels. (**B**) The analysis of the PBMCs from patient 2 before (in red) and after (in purple) in vivo treatment with 63.9 µg/die of LT3, given orally for one week. The profiles of oxygen consumption and extracellular acidification rates are reported in the upper panels; the graphs showing basal and maximal OCR and spare capacity are reported in the lower panels. OCR was measured continuously throughout the experimental period at baseline and in the presence of the indicated drugs: 1 μM oligomycin, 1 μM FCCP, and 1 μM rotenone with 1 μM antimycin A (R + A).

Mitochondrial respiration, measured by Seahorse, indicated a marked dysfunction at T0 in both patients. Both basal and maximal OCR were severely reduced, and ECAR was markedly hampered. We repeated the analysis on PBMCs of patient 1 after in vitro treatment with LT3 at 100 nM for 1 h and we observed a significant improvement in the basal (two-fold increase) as well as in the maximal OCR (1.4-fold increase) (Figure 5A). A more evident effect on mitochondrial respiration was observed when we examined the effects of LT3 on the PBMCs of patient 2 after in vivo treatment of the patient with LT3 given orally at 63.9 µg/die for 1 week. Seahorse analysis of in vivo T3-treated PBMCs demonstrated a clear and evident stimulation of the mitochondrial respiration in patient 2 (Figure 5B). We observed a significant increase in the basal (2.7-fold increase) and in the maximal OCR (3.5-fold increase). The ECAR was stimulated by LT3 treatment too. We observed also an improvement in the mitochondrial spare respiratory capacity percentage (1.3-fold increase). Taken together, our data regarding both the in vitro and in vivo effects of LT3 treatment, indicate that LT3 can restore a normal mitochondrial respiration on PBMCs of our COVID-19 patients with NTIS.

**Figure 6:**
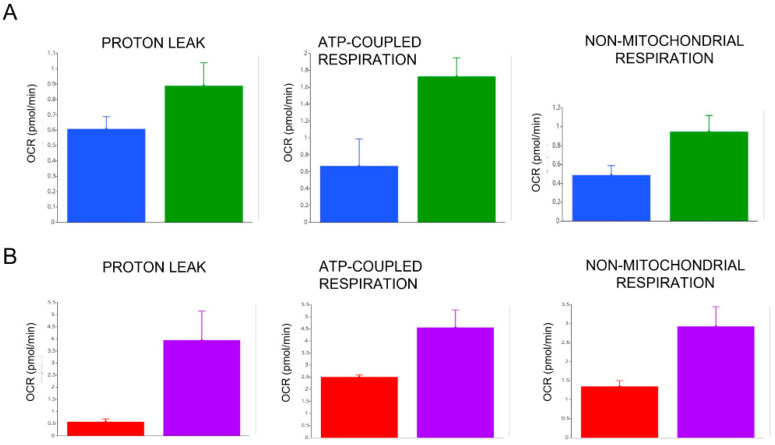
Mitochondrial respiration in two COVID-19 patients in the pilot study. Seahorse analysis of proton leak, ATP-produced respiration and non-mitochondrial respiration, performed on PBMCs obtained from COVID-19 patients with NTIS and treated in vivo and in vitro with LT3. (**A**) Analysis of the PBMCs from patient 1 before (in blue) and after (in green) in vitro treatment with 100 nM of LT3 for 1 h. (**B**) analysis of the PBMCs from patient 2 before (in red) and after (in purple) in vivo treatment with 63.9 µg/die of LT3, given orally for one week.

An increase of other mitochondrial respiration parameters, including proton leak, ATP-produced respiration and non-mitochondrial respiration, was observed upon treatment with LT3, given both in vitro at 100 nM for 1 h (Figure 6A) and in vivo at 63.9 µg/die for one week (Figure 6B).

These results confirm those obtained in our cohort of COVID-19 patients and reinforce the role of T3 in regulating mitochondrial respiration and, in particular, in stimulating both the maximal respiratory capacities and proton leak. 

## 4. Discussion

Oxidation is a process that occurs naturally in the body when oxygen combines with reduced molecules and provides energy. Any process that stimulates energy production is associated with an increase in oxidation and results in the generation of ROS. An efficient scavenger and antioxidant system is required to reduce all the excessive ROS generated. Oxidative stress is represented by the imbalance between the generation of reactive oxygen/nitrogen species and the ability to counteract them with an efficient antioxidant capacity. Oxidative stress and inflammation play key roles in the multisystem disorder caused by the SARS-CoV-2 infection, and they have been linked to many different pathologies that are predisposed to critical outcomes in COVID-19, including cardiovascular disease and diabetes mellitus type 2 [32,33]. In addition, the uncontrolled oxidative stress produced by the SARS-CoV-2 infection is associated with a pro-inflammatory state and cytokine production, which may contribute to the development of a respiratory syndrome, and in the worst cases, may lead to death. Considering the pathogenic role of oxidative stress, the use of antioxidation therapy has been recently proposed in a clinical trial to prevent organ and tissue damage triggered by the cytokine storm [34].

The clinical severity of COVID-19 correlates with a dysfunction of the immune system. Two major hallmarks of the disease are represented by the occurrence of lymphopenia together with the induction of a peculiar systemic hyper-inflammation condition, known as a “cytokine storm” or macrophage activation syndrome (MAS) [35,36]. Mitochondrial dysfunction represents a key factor in the severity of the COVID-19 disease. It contributes to all known COVID-19-severity risk factors, such as aging [37,38] and various age-related metabolic diseases, including obesity [39], metabolic syndrome [40,41], diabetes [42], hypertension [43], and coronary heart disease [44]. It also plays a relevant role in other risk factors, such as lung diseases, cancers and neurodegenerative diseases [45,46]. In agreement with these observations, an altered bioenergetic and mitochondrial dysfunction has been recently described in immunological circulating blood cells obtained from patients with COVID-19 pneumonia. Mitochondrial function and associated metabolic changes were recently analyzed in live cells from patients with COVID-19. In a study, based on a cohort of seven COVID-19 patients, it was demonstrated that their PBMCs exhibited reduced maximal respiration and reserve capacity, indicating compromised mitochondrial respiration or mitochondrial dysfunction, as compared to nine healthy controls and seven patients with other chest infections [47]. In this study, there was no mention regarding the thyroid function and the possible occurrence of NTIS in these patients. In another cohort of five COVID-19 patients and seven matching healthy controls, the same analysis was performed on CD4+ T cells. This study demonstrated an altered cell proliferation but not mitochondria functionality, measured as the mitochondrial oxygen consumption rate (OCR) and extracellular acidification rate (ECAR) [48]. The mitochondrial functional respiration was recently evaluated in monocytes isolated from PBMCs of 13 COVID-19 patients with COVID-19 pneumonia [49]. Monocytes from COVID-19 patients displayed a reduction in basal and maximal respiration, proton leak, and spare respiratory capacity, compared to those from the healthy controls. Finally, an analysis of eight single-cells RNA-seq datasets, obtained from PBMCs, nasopharyngeal samples, and bronchoalveolar lavage fluid (N = 1,192,243 cells), revealed a significantly reduced mtDNA gene expression in immune system cells in COVID-19 patients [50]. All together, these results indicate that the mitochondrial metabolic pathway is altered in COVID-19 patients and suggest that targeting this pathway could represent a novel strategy for COVID-19 treatment. 

There is much evidence in the literature, indicating that THs regulate cell bioenergetics by acting through direct effects on mitochondrial function [25,51]. Mitochondria have been shown to be the major sites of T3 accumulation in cells, where they exert a direct effect on mitochondrial activity and on energy metabolism [52]. In addition, free radical production is associated with the hypermetabolic state in hyperthyroidism, whereas the hypometabolic state induced by hypothyroidism leads to a decrease in free radical production [30,53,54].

Recently, we have demonstrated that PBMCs obtained from COVID-19 patients with NTIS showed a peculiar gene expression signature [16]. In particular, we found four genes that were deregulated in these patients, namely the IFIT3, the NLRP3, the CD38, and the CD79B. All these genes encode for proteins involved in the mitochondrial function. The IFIT3 is localized in the mitochondria and is significantly induced upon RNA virus infection. It plays a relevant role as a modulator of innate immunity [55]. The NLRP3 inflammasome is triggered by a variety of situations of host ‘danger’, including infection. It can sense mitochondrial dysfunction, and it is activated upon mitochondrial damage and destabilization [56,57]. In addition, it can sense mitochondrial dysfunction, and it is activated upon mitochondrial damage and destabilization [58,59]. The CD38 can drive mitochondrial trafficking in multiple myeloma [60], as well as after stroke [61]. In addition, it is involved in age-related NAD decline and mitochondrial dysfunction [62]. Finally, the CD79B interacts with Syk, which promotes mitochondrial superoxide generation via the modification of the mitochondrial electron transport chain in hematopoietic and nonhematopoietic cells [63], and its phosphorylation and subsequent translocation to lipid rafts appears to be involved in the generation of reactive oxygen species (ROS), a decrease in mitochondria membrane potential, and the induction of caspase-dependent apoptosis [64]. 

Seahorse XF technology proved to be capable of assessing, in a highly sensitive manner, the mitochondrial function in terms of several bioenergetic parameters, including basal respiration, ATP production, proton leak, maximal respiration, spare respiratory capacity, and non-mitochondrial respiration [65,66]. The assessment of the bioenergetic profile of human PBMCs has emerged as a new translational research biomarker and a new potential tool for the assessment of organ-specific mitochondrial dysfunction, relevant for the clinical outcome of critical illness [67]. Functional mitochondrial changes have been recently analyzed using Seahorse technology in ex vivo PBMCs obtained from patients with COVID-19 [47,48,49]. It has been demonstrated that SARS-CoV-2 is able to hijack host mitochondrial function and manipulate metabolic pathways for its own advantage. As a result, the PBMCs are left with a compromised mitochondrial function and an energy deficit that can be compensated for by a metabolic switch to glycolysis [49]. In another study, the Seahorse XFe24 analyzer was used to demonstrate the effect on neutrophils metabolism of COVID-19 patients, measured as ECAR, of the combined treatment with lipopolysaccharide and colchicine [68]. In disagreement with the previous study, a very recent observation, based on the analysis by high-resolution respirometry using the Oxygraph O_2_k (OROBOROS Instruments, Innsbruck, Austria) and by the Cellular Oxygen METabolism (COMET) monitor, found an increase in mitochondrial oxygen tension (mitoPO_2_) and mitochondrial oxygen consumption (mitoVO_2_) between the isolated PBMCs from the SARS-CoV-2 patient groups, as compared to those from healthy controls and those patients without COVID-19 undergoing general anesthesia because of cardiothoracic surgery [69]. According to these results, mitochondrial respiration is increased in cases of severe COVID-19 compared to other critically ill patients, suggesting the occurrence of a relative hypermetabolic state in such patients. On the contrary, our results indicate that mitochondrial respiration is indeed hampered from COVID-19, and this effect was more evident in those with NTIS. Moreover, we demonstrated that treatment with LT3, either in vitro or in vivo, was able to stimulate mitochondrial respiration in PBMCs or our COVID-19 patients with NTIS. Treatment with LT3 in our patient 2 was also able to ameliorate the hydroelectrolytic alterations observed at admission and measured by BIA, while further reduction in serum FT3 levels, observed in patient 1, was associated with a worsening of BIA parameters and, in particular, on the hydration of fat-free mass, confirming our previous observation that T3 plays a crucial role in the control of hydroelectrolytic balance at the periphery [17]. In our patient 1, affected by severe COVID-19 with NTIS, treatment of the PBMCs in vitro with LT3 resulted in a significant increase in mitochondrial respiration, measured as basal and maximal OCR. A more evident effect was seen in the PBMCs of the patient 2 who received in vivo oral treatment with LT3 for one week, suggesting that LT3 exerts a stimulatory effect on OCR, associated to mitochondrial respiration and on ECAR, ascribed to glycolytic capacity. In addition, LT3 treatment ameliorates the spare respiratory capacity, which indicates the capability of the cells to respond to an increased energetic demand. It is interesting to note that, since mitochondrial respiration of PBMCs of our COVID-19 patients was stimulated after acute in vitro treatment with LT3 for 60 min (Figure 6A), we may speculate that LT3, besides its well-known transcriptional activity, may also act through other non-transcriptional effects. To the best of our knowledge, the stimulating effect of T3 on mitochondrial respiration in PBMCs observed in our COVID-19 patients with NTIS has never been reported before. One limit of our study relies on the fact that we did not perform sorting and analysis of the different cell populations within the PBMC fraction and, therefore, we could not confirm previous observations regarding the importance of monocytes in COVID-19 immunopathogenesis [49] or assess whether they could represent specific targets of TH action in these patients.

Since severe COVID-19 progression can be divided into phases, including early infection, host immune response, hyperinflammatory phase, and multiorgan dysfunction [70], we cannot exclude that the discrepancies between our results and those reported by Streng et al. [69] could be due to a different timing of patient inclusion, or alternatively, to the different methods used to analyze mitochondrial respiration. Our results regarding the stimulatory effects of LT3 on mitochondrial respiration are in accordance with those previously reported in other conditions and in different types of cells, including murine brown adipocytes [71] and murine, as well as human alveolar epithelial cells [72]. 

The two major differences we observed between PBMCs obtained from our cohort of COVID-19 patients, with and without NTIS, with regard to mitochondrial respiration, are related to the maximal respiration capacity and the proton leak. Both parameters, measured by Seahorse, were reduced in COVID-19 patients with NTIS compared to those without NTIS. The maximal OCR is measured by adding the uncoupler FCCP which acts by mimicking a physiological “energy demand” to meet an upcoming metabolic stress challenge. The induced stress stimulates the respiratory chain of the cells to reach the maximum rate of respiration they can achieve with a consequent activation of the rapid oxidation of substrates (sugars, fats, and amino acids). The proton leak is measured after the injection of an ATP synthase inhibitor (oligomycin) and reflects the remaining basal respiration not coupled to ATP production. Proton leak through uncoupling proteins (UCPs) is responsible for most of the uncoupling respiration in mitochondria, which generates heat instead of ATP, a TH effect typically observed in brown adipose tissue and muscle [28]. It has been reported that T3 is able to modulate both a basal and inducible proton leak in the mitochondria of skeletal muscle cells [73]. Proton leak has been considered as a sign of mitochondrial damage. However, according to the “uncoupling to survive” hypothesis, proton leak pathways are aimed to minimize oxidative damage by tempering electrical potential (Δp) and mitochondrial superoxide production [74]. The proportion of respiration that is used to drive the energy-dissipating “futile” proton cycle across the mitochondrial inner membrane is surprisingly high. It has been calculated, in fact, that the energy entirely devoted to driving this cycle accounts for 20–25% of the cellular basal metabolic rate. Such a proportion is remarkably constant even in species of widely different body mass, ranging from a mouse to a horse [75]. Since the mitochondrial proton cycle appears to be such an important energy drain and a wide range of organisms are prepared to pay a very high energetic price to maintain it, the functions that depends on it must be very important. Among such functions, could be several possible relevant ones, including thermogenesis, an improved ability to regulate energy metabolism, a safety valve for the avoidance of dielectric breakdown of the membrane at excessive membrane potentials, the ability to continue carbon metabolism when the ATP demand is low, the regulation of body mass, and the attenuation of free radical production [74]. Reduced proton leak observed in our COVID-19 patients with NTIS, strictly dependent on the reduction of FT3 serum levels, could be part of the adaptive response aimed to spare energy. However, it is reasonable to hypothesize that such an adaptive response may be associated with the induction of several associated cellular damages. The crucial role played by THs in such regulation is also demonstrated by experiments that were performed many years ago on liver mitochondria isolated from rats. These experiments demonstrated that the thyroid status of the animals strongly correlated with the basal proton conductance in mitochondria and, in particular, with the increase in the proton conductance of the inner mitochondrial membrane [76]. Our results agree with such observations and reinforce the hypothesis that thyroid hormones control the respiration rate required to balance the backflow of protons across the inner mitochondrial membrane.

In summary, our results indicate that thyroid hormones, in particular the active form LT3, play a relevant role in controlling mitochondrial respiration, energy production, and oxidative stress in circulating the immune cells of our COVID-19 patients. Based on the reduced serum levels of dROMs, and on the positive balance between oxidation and anti-oxidation, we support the hypothesis that reduced levels of serum FT3, observed in COVID-19 patients with NTIS, may exert a beneficial effect on the course of the disease. The reduced generation of ROS and reduced induction of oxidative stress exerts, in fact, a protective role in the pathogenesis of the acute immunological damages caused by SARS-CoV-2 infection. Since the reduced levels of FT3 are associated with reduced oxidation, it is possible that the use of T3 treatment, proposed for the management of severe COVID-19 [77], could be responsible for an increase in oxidative stress. The use of antioxidants, such as N-acetylcysteine, proved to be effective against severe COVID-19 [78] and could be useful to mitigate such effects. 

However, the beneficial effect observed in COVID-19 patients with NTIS comes with a counterpart, represented by the consequence of reduced mitochondrial respiration. Such a negative effect is detrimental to the cells, since it is responsible for a markedly reduced production of energy generated by the circulating immune cells, thus dampening their response to viral infection. 

It has been reported that exogenous nitric oxide (NO) can reduce systemic hyperinflammation and oxidative stress in COVID-19 patients [79]. Moreover, thyroid status could influence both the formation of and response to NO by the arterial vessels in rats [80]. It has been speculated that, since it was reported that NO administration is also able to improve arterial oxygenation, and to restore pulmonary alveolar cellular integrity, such administration, especially in the early stages of the disease, could have a preventive effect on lung damage in the management of COVID-19 patients [80]. However, we were not able to measure NO levels or perform other antioxidant assays besides the BAP test in our samples because of an insufficient amount of blood left after the measurements performed for the study. Another limit of our study was the small number of COVID-19 patients recruited for the study. It was designed as a pilot study and the results obtained will be useful to plan the clinical trial that we will perform and that will be focused on the potential use of THs, and especially T3, in critically ill patients admitted to the ICU. 

## 5. Conclusions

Our results indicate that reduced serum FT3 levels, observed in COVID-19 patients with NTIS, have an opposite effect on oxidative balance and on mitochondrial respiration. On one side, the acute reduction of serum FT3 levels is responsible for the reduced generation of ROS, with a consequential reduction in the activation of the pathophysiological processes involved in the progression of COVID-19, including cytokine production, inflammation, and cell death. However, this beneficial adaptative effect comes with a counterpart that is represented by reduced mitochondrial respiration and a consequential reduction in energy production. This hypoenergetic state of immune cells induced by NTIS may compromise their reparative capacity and their ability to react to the damages induced by SARS-CoV-2 infection. Our pilot study, performed on two COVID-19 patients with NTIS, reinforced the role of T3 in regulating mitochondrial respiration and suggests that treatment with T3 could be beneficial in ameliorating mitochondrial respiration in circulating immune cells. However, the demonstration of the benefit of such treatment in NTIS-complicating COVID-19, as well as other critical illness, needs to be confirmed in future interventional, randomized clinical trials, with specific outcomes focused on mitochondrial function.

## Figures and Tables

**Table 1:** Epidemiological and clinical characteristics of COVID-19 patients.

PATIENTS’S CHARACTERISTICS	ALL (*n* = 54)	LOW FT3 (*n* = 35)	VERY LOW FT3 (*n* = 9)	NORMAL FT3 (*n* = 10)
Age	68 (29–93)	69 (29–93)	67 (29–93)	69 (29–93)
Sex				
M	37 (69%)	22 (63%)	7 (78%)	8 (80%)
F	17 (31%)	13 (37%)	2 (22%)	2 (20%)
Comorbidity	34 (63%)	25 (71%)	5 (56%)	4 (40%)
Diabets	5 (9%)	3 (9%)	1 (11%)	1 (10%)
Hypertension	21 (39%)	15 (43%)	3 (33%)	3 (30%)
Heart disease	15 (28%)	12 (34%)	3 (33%)	0 (0%)
Obesity	4 (7%)	1 (3%)	2 (22%)	1 (10%)

**Table 2:** Epidemiological and clinical characteristics of two COVID-19 patients in the pilot study.

PTS	GENDER	AGE (yrs)	HEIGHT (cm)	WEIGHT (Kg)	BMI (Kg/m^2^)	SOFA Score	TIME	FT3 (pg/mL)	FT4 (ng/dL)	TSH (µlU/mL)	THERAPY
**#1**	M	58	170	75	26	5	T0	1.3	1	0.16	Dexamethasone (4 mg OD)
							T1	1	0.9	0.07	
**#2**	F	53	170	85	29.4	2	T0	1	0.9	0.39	Dexamethasone (6 mg OD) + Liothyroinine (21.3 µg TID)
							T1	1.8	0.7	0.03	

**Table 3:** Oxidative Stress in COVID-19 patients with and w/o NTIS.

	VERY LOW FT3 (<1.0 pg/mL)	LOW FT3 (>1.0 ≤ 1.7 pg/mL)	NORMAL FT3 (>1.8 pg/mL)	*p* VALUES (VERY LOW VS. NORMAL)
**dROMs (U-CARR)** **medium values (** **±** **SD)**	376.5 (±44.6)	395.0 (±55.8)	449.5 (±62.1)	*p* < 0.005
**BAP (** **µmol/L)** **medium values (** **±** **SD)**	2068.4 (±370.2)	1680.8 (±682.6)	1605.4 (±690.4)	*p* < 0.05
**OSi Index (DROMs/BAP)** **medium values (** **±** **SD)**	0.18 (±0.06)	0.23 (±0.03)	0.27 (±0.1)	*p* < 0.005

## Data Availability

The datasets used and/or analyzed during the current study are available from the corresponding author on reasonable request. All data generated or analyzed during this study are included in this published article.

## Supplementary Materials

The following supporting information can be downloaded at: https://www.mdpi.com/article/10.3390/antiox11101998/s1, **Figure S1**. Lethality rate among COVID-19 patients with and w/o NTIS; **Figure S2**—Mitochondrial respiration in COVID-19 patients compared to Healthy Donors. Medium traces of OCR of PBMCs from COVID-19 patients with and w/o NTIS and of healthy donors.

## Abbreviations

COVID-19: coronavirus disease 2019; TH: thyroid hormone; T3: 3,5,3′-triiodothyronine; T4: thyroxine; FT3: free triiodothyronine; LT3: liothyronine; LT4: levothyroxine; NTIS: nonthyroidal illness syndrome; ESS: euthyroid sick syndrome; d-ROMs: derivatives of reactive oxygen metabolites; BAP: biological antioxidant potential; OSi: oxidative stress index; SOFA score: sequential organ failure assessment score; PBMCs: peripheral blood mononuclear cells; OCR: oxygen consumption rate; ECAR: extracellular acidification rate; BIA: bioelectrical impedance analysis; Rz: Resistance; Xc: Reactance; PhA: Phase angle; Na*_e_*:K*_e_*: Na:K exchange rate; BCM: body cell mass; BMI: body mass index; TBW: total body water; ECW: extracellular water; ICW: the intracellular water; FFM: fat-free mass; FM: fat mass.
