# Supplementary material for: Opposite Effect of Thyroid Hormones on Oxidative Stress and on Mitochondrial Respiration in COVID-19 Patients
Source: Antioxidants (Basel). 2022 Oct 8;11(10):1998. doi: 10.3390/antiox11101998 (PMC9598114; doi:10.3390/antiox11101998)
Supplement: Supplementary file 1 [file antioxidants-11-01998-s001.zip › antioxidants-1900893-supplementary.pdf]

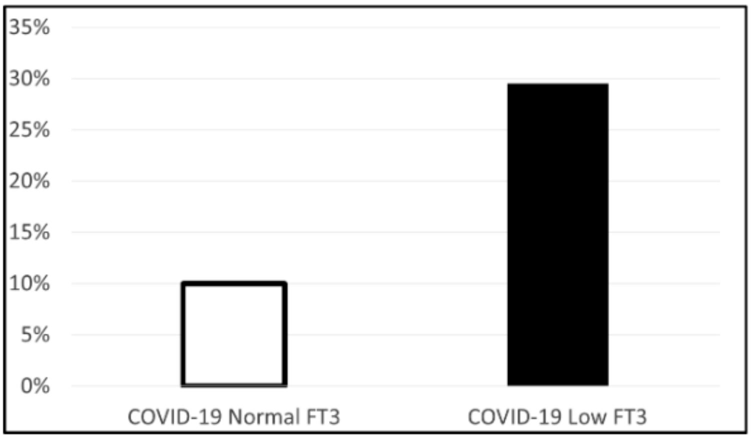

Figure S1. Lethality rate among COVID-19 patients with and w/o NTIS.

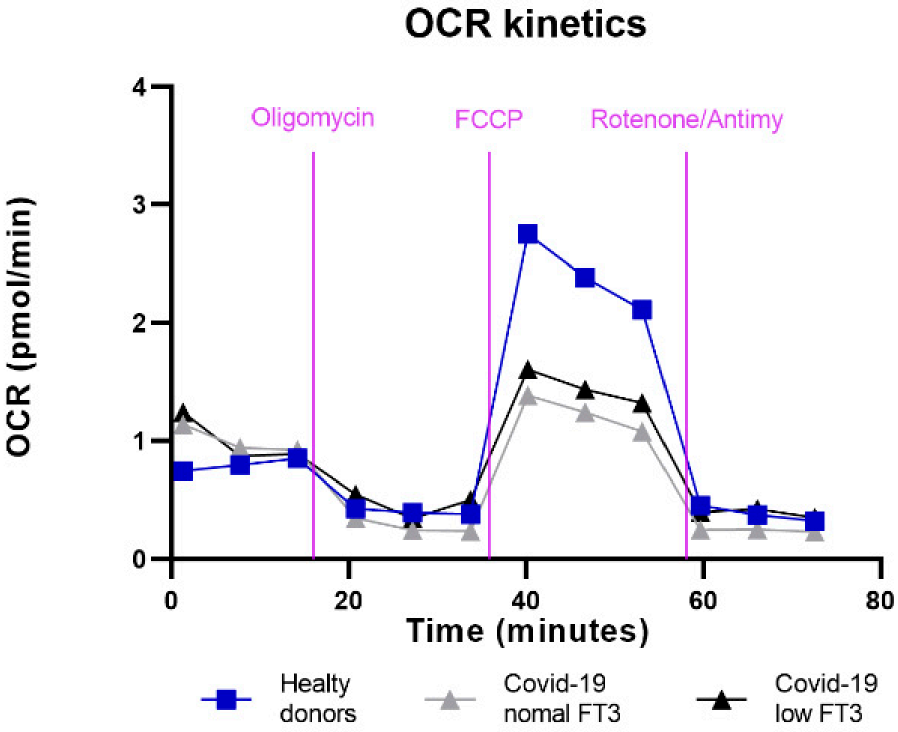

Figure S2. Mitochondrial respiration in COVID-19 patients compared to Healthy Donors. Medium traces of OCR of PBMCs from COVID-19 patients with and w/o NTIS and of healthy donors.
